# Prevalence of pediatric delirium among critically ill children: a meta-analysis of observational studies

**DOI:** 10.3389/fped.2026.1888789

**Published:** 2026-07-22

**Authors:** Xin-Mao Shi, Jie Hu, Yu Chen, Chun-Xia Wang, Zheng-Kui Zhou, Mei Feng

**Affiliations:** 1 Department of Critical Care Medicine, West China Hospital/West China School of Nursing, Sichuan University, Chengdu, China; 2 Department of Gastroenterology, The Third People's Hospital of Chengdu, Clinical College of Southwest Jiao Tong University, The Affiliated Hospital of Southwest Jiaotong University, Chengdu, China

**Keywords:** critically ill children, epidemiology, meta-analysis, pediatric delirium, prevalence

## Abstract

**Background:**

Pediatric delirium (PD) is a serious neurological complication in critically ill children, associated with prolonged hospitalization, extended mechanical ventilation, and increased mortality. However, the pooled prevalence of PD in this population remains unclear. This meta-analysis of observational studies aimed to determine the overall prevalence of PD among critically ill children and to identify its potential moderating factors.

**Methods:**

A comprehensive literature search was conducted in PubMed, Web of Science, Scopus, Embase, Cochrane Library, and CINAHL from inception to May 26, 2026. Methodological quality was appraised with the Joanna Briggs Institute (JBI) critical appraisal checklist for prevalence studies. Pooled prevalence of PD was calculated utilizing a DerSimonian-Laird random-effects model. To stabilize variances and improve the normal approximation, a logit transformation was applied for the meta-analysis of prevalence data. Heterogeneity was assessed using Cochran's *Q* test and quantified with the I² statistic. Subgroup analyses and meta-regression were conducted to explore potential sources of heterogeneity.

**Results:**

This meta-analysis included 40 observational studies, involving 18,875 critically ill children. The pooled prevalence of PD among critically ill children was 38.1% (95% CI: 32.5–43.8%, I^2^ = 98.7%, 95% PI: 3.6–72.4%). According to the JBI critical appraisal tool, 11 studies (27.5%) were rated as having a low risk of bias, 26 (65.0%) a moderate risk, and 3 (7.5%) a high risk. Subgroup analysis revealed significant variation in pooled prevalence across different assessment tools. Meta-regression suggested that prevalence was not significantly associated with sample size, income status, study design, or assessment tool.

**Conclusions:**

Our findings indicate that PD is a prevalent neurological complication among critically ill children. However, due to extremely high heterogeneity and a very wide prediction interval, this pooled estimate should not be interpreted as a stable universal figure, but merely as a summary of highly heterogeneous studies. Therefore, caution is warranted when generalizing the pooled estimate to local settings.

**Systematic Review Registration:**

identifier CRD420261339560.

## 1 Introduction

Pediatric delirium (PD) is an acute neuropsychiatric syndrome characterized by attention deficits and alterations in consciousness (1, 2). Critically ill children are generally vulnerable to experiencing delirium due to several risk factors, including exposure to sedative and analgesic medications, environmental stressors such as sleep deprivation and sensory overload, as well as immature brain functioning (3, 4). As a common complication of critical illness, PD is associated with various adverse outcomes in critically ill children, including prolonged hospitalization, increased duration of mechanical ventilation, elevated risk of nosocomial infections, and a significantly higher mortality rate (5–8). Furthermore, the clinical presentation of PD in critically ill children often diverges from that observed in adults, featuring irritability, withdrawal, or inconsolability, which frequently leads to misinterpretation as behavioral problems or emotional distress (9). Therefore, the prevalence and burden of PD in critically ill children have long been underestimated, highlighting the need for a comprehensive synthesis of global data to guide clinical management.

In recent years, there has been growing interest in the epidemiology of PD among critically ill children. The development of standardized assessment tools, including the Cornell Assessment of Pediatric Delirium (CAPD) and the pediatric Confusion Assessment Method for the ICU (pCAM-ICU), has facilitated breakthrough advances in the epidemiology of PD in critically ill children. The widespread use of these instruments has enabled routine bedside screening across all pediatric age groups, thereby allowing a more precise characterization of the epidemiological landscape of PD. However, considerable heterogeneity has been observed in the reported prevalence of PD among critically ill children, with estimates varying from 7.7% to 83.8% across studies (10–13). This heterogeneity may be explained by variations in study populations, diagnostic tools, geographic locations, and management strategies across studies. Specifically, the predominance of single-center designs and studies focused on specific subgroups in the existing literature limits the generalizability of findings. In addition, heterogeneity in the screening tools and diagnostic criteria used across studies has contributed to substantial variability in reported prevalence rates. Although a recent systematic review has provided a pooled prevalence estimate of PD in critically ill children, it is subject to several limitations (14). First, a substantial number of the included studies were primarily designed for the development and validation of standardized assessment tools. In such studies, prevalence was considered a secondary outcome rather than the primary endpoint, and therefore, the study designs may not have adequately accounted for confounding factors, potentially compromising the accuracy of prevalence estimates. Moreover, neither sensitivity analysis nor publication bias assessment was conducted, compromising the reliability of the pooled estimates. Finally, the availability of newly published prospective studies warrants an update to the prior pooled estimates. More importantly, a comprehensive synthesis integrating global data on the prevalence of PD in critically ill children across various geographic regions and healthcare resource settings is absent. Therefore, this meta-analysis aimed to determine the pooled prevalence of PD in critically ill children and identify its potential moderating factors.

## 2 Methods

This meta-analysis was conducted in accordance with the Preferred Reporting Items for Systematic Reviews and Meta-Analyses (PRISMA) guidelines (15) and was registered with PROSPERO (CRD420261339560).

### 2.1 Search strategy

Literature searches were conducted in PubMed, Web of Science, Scopus, Embase, Cochrane Library, and CINAHL from inception to May 26, 2026. The search strategy employed Boolean operators to combine MeSH terms and keywords, such as critically ill children, critically ill pediatric patients, pediatric intensive care units, delirium, pediatric delirium, and prevalence. To ensure comprehensiveness, we also examined the reference lists of all included studies for additional eligible articles. Detailed search strategies are provided in Supplementary Table S1.

### 2.2 Study selection and data extraction

Studies were included if they: (1) involved critically ill children (< 18 years); (2) had a cross-sectional or cohort design; (3) used a validated tool to assess PD; (4) reported or allowed calculation of PD prevalence. Furthermore, we excluded conference abstracts, protocols, case reports, and reviews, as well as studies not published in English. For duplicate publications, we retained only the study with the largest sample size for analysis. To ensure conceptual consistency before pooling, we extracted only the baseline point prevalence for cohort studies.

Data extraction was performed independently by two reviewers using a standardized form. Any discrepancies were resolved through consultation with a third reviewer to reach a consensus. The following information was extracted: first author, publication year, country, study design, sample size, age, proportion of females, assessment tool, and prevalence of PD.

### 2.3 Methodological quality assessment

Two reviewers independently evaluated the methodological quality of each included study using the Joanna Briggs Institute (JBI) critical appraisal checklist for prevalence studies (16). The tool assesses nine domains, with each item rated as “yes” (low risk), “no” (high risk), or “unclear/not applicable.” Studies were subsequently categorized as having low (≥ 70% “yes” scores), moderate (50%–69% “yes”), or high (≤ 49% “yes”) risk of bias. Discrepancies between reviewers were resolved by discussion with a third reviewer until consensus was achieved.

### 2.4 Data analysis

Data analyses were conducted with Stata 15.0. A DerSimonian-Laird random-effects model was used to calculate the pooled prevalence of PD in critically ill children and its corresponding 95% confidence interval (CI). To stabilize variances and improve the normal approximation, a logit transformation was applied for the meta-analysis of prevalence data. Heterogeneity among studies was quantified using the I² statistic, with a value of 50% or greater indicating substantial heterogeneity (17). Additionally, Cochran's *Q* test was performed to formally assess heterogeneity, and a *p*-value less than 0.10 was considered statistically significant. We calculated the 95% prediction interval (PI) to provide a range for the true prevalence in future similar settings. Subgroup analyses were conducted according to study design, income status, and assessment tool to determine potential sources of heterogeneity. Furthermore, univariate meta-regression analyses were conducted to assess the association between covariates (sample size, study design, income status, and assessment tool) and the estimated prevalence. Country income status was classified using the World Bank's fiscal year 2026 classification. Publication bias was examined using funnel plots and Egger's test. To test the robustness of the findings, we performed a leave-one-out sensitivity analysis, as well as additional analyses excluding high risk of bias studies, very small studies (*n* < 100), cross-sectional studies, and non-CAPD studies. Statistical significance was defined as two-sided *P* < 0.05.

## 3 Results

### 3.1 Study selection and characteristics

**Figure 1:**
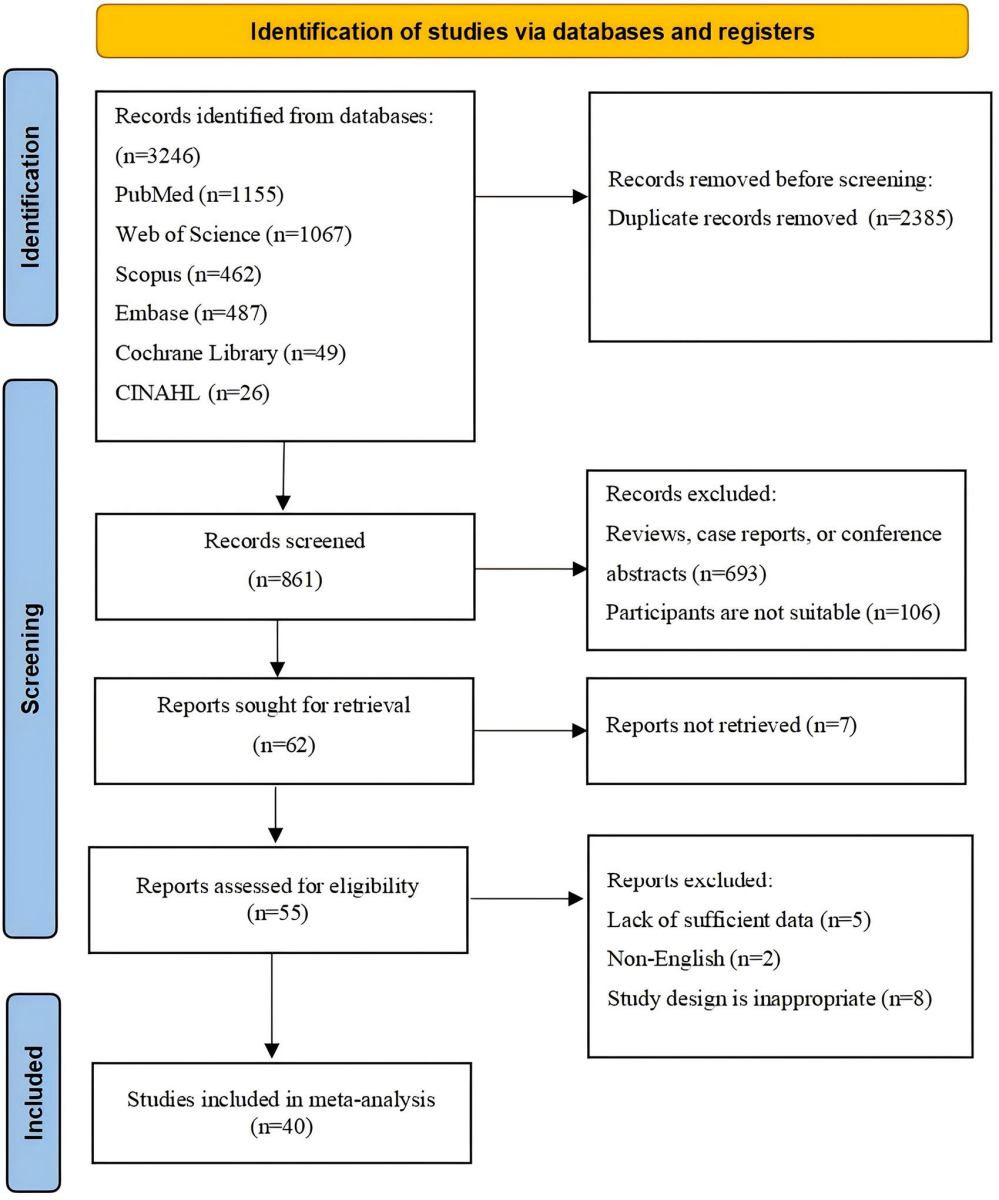
Flow diagram of study selection.

The database search initially identified 3,246 records. After removing duplicates, 861 records were screened by title and abstract. Of these, 799 records were excluded, and the remaining 55 full-text articles were assessed for eligibility. Following full-text review, 15 articles were excluded for various reasons (Supplementary Table S2), leaving 40 studies that met all inclusion criteria and were included in the meta-analysis. The detailed study selection process is summarized in Figure 1.

A total of 18,875 critically ill children from 40 studies were included. The studies were conducted across 11 countries: the USA (*n* = 21), China (*n* = 6), Colombia (*n* = 3), Germany (*n* = 2), India (*n* = 2), and one study each from Brazil, Canada, Iran, Pakistan, Saudi Arabia, and Turkey. Sample sizes ranged from 26 to 2,446 participants. Furthermore, most studies assessed PD using the Cornell Assessment of Pediatric Delirium. The reported prevalence of PD varied considerably across studies, ranging from 7.7% to 83.8%. The detailed characteristics of the included studies are presented in Table 1.

**Table 1:** Characteristics of included studies.

Study	Country	Study design	Sample size	Age	Female (%)	Assessment tool	Prevalence (%)
AlDaithan et al. (1)	Saudi Arabia	Cohort	890	NA	45.2	CAPD	69.4
Alvarez et al. (2)	USA	Cohort	99	NA	47.5	CAPD	56.6
Cano et al. (3)	Colombia	Cross-sectional	26	5–12 years	42.3	pCAM-ICU	57.7
Christian et al. (4)	USA	Cohort	43	NA	58.1	CAPD	67.4
Dechnik et al. (18)	USA	Cohort	734	NA	42.0	CAPD	37.3
Dervan et al. (5)	USA	Cohort	2,446	NA	46.9	CAPD	44.4
Dervan et al. (19)	USA	Cohort	534	1.4 years	43.4	CAPD	44
Flores et al. (10)	Brazil	Cohort	65	47.62 ± 58.11 months	53.9	CAPD	7.7
Frost et al. (6)	USA	Cohort	282	19 months	37.2	psCAM-ICU	45.0
Fu et al. (11)	China	Cross-sectional	160	28 months	42.5	CAPD	83.8
Gray et al. (12)	USA	Cohort	149	0.3–7 years	46.3	CAPD	28.9
Gupta et al. (13)	USA	Cohort	95	NA	44.2	CAPD	20.0
Henao-Castaño et al. (20)	Colombia	Cohort	52	33.3 ± 18.1 months	55.8	psCAM-ICU	71.1
Koth et al. (21)	USA	Cohort	942	NA	43.8	CAPD	67.3
Lachman et al. (22)	USA	Cohort	102	11.1 ± 3.7 years	58.3	pCAM-ICU	37.3
Lei et al. (23)	China	Cohort	1,576	12 months	41.1	CAPD	26
Leng et al. (24)	USA	Cohort	138	5 years	46.4	CAPD	37
Lin et al. (25)	China	Cohort	501	12 months	50.5	CAPD	58.7
Mao et al. (26)	China	Cohort	330	19.27 ± 19.76 months	49.1	CAPD	26.1
Meyburg et al. (27)	Germany	Cohort	93	3.5 ± 4.7 years	49.5	CAPD	65.6
Mirzaaghayan et al. (28)	Iran	Cross-sectional	92	25.00 ± 32.70 months	48.9	CAPD	37
Motwani et al. (29)	India	Cross-sectional	476	NA	44.4	CAPD	20.2
Nashit et al. (30)	Pakistan	Cross-sectional	201	5.14 ± 4.03 years	38.3	CAPD	24.9
Patel et al. (31)	USA	Cohort	194	NA	41.2	CAPD	49.0
Pilarz et al. (32)	USA	Cohort	1,865	0.32 years	44.9	CAPD	25.6
Pilato et al. (33)	USA	Cohort	1,145	NA	43.3	CAPD	14.1
Ricardo et al. (34)	Colombia	Cross-sectional	156	11	47.4	pCAM-ICU	18.6
Schumann et al. (35)	Germany	Cohort	293	NA	NA	CAPD	40.3
Siegel et al. (36)	USA	Cross-sectional	147	NA	NA	CAPD	22.4
Silver et al. (37)	USA	Cohort	207	13 momths	41.5	CAPD	27.1
Smith et al. (38)	USA	Cohort	300	20 months	37.3	psCAM-ICU	41.3
Smith et al. (39)	USA	Cohort	224	NA	NA	CAPD	55.4
Sudhakar et al. (40)	India	Cohort	105	11.10 ± 3.28 years	31.4	pCAM-ICU	14.3
Thibault et al. (41)	Canada	Cohort	179	NA	41.3	CAPD	65.4
Traube et al. (42)	USA	Cross-sectional	835	NA	46.0	CAPD	25
Traube et al. (43)	USA	Cohort	1,547	NA	43.1	CAPD	17.3
Weatherly et al. (44)	USA	Cohort	148	7 months	42.6	pCAM-ICU	23.6
Xu et al. (45)	China	Cohort	315	65.3 ± 54.3 months	43.8	CAPD	29.2
Xu et al. (46)	China	Cohort	1,047	68.92 ± 53.03 months	43.0	CAPD	26.6
Yontem et al. (47)	Turkey	Cohort	142	43 months	40.1	CAPD	9.9

### 3.2 Methodological quality assessment results

The methodological quality of the included studies is summarized in Supplementary Table S3. Of the 40 assessed responses (Table 2), 11 (27.5%) were considered to have a low risk of bias, 26 (65.0%) a moderate risk, and 3 (7.5%) a high risk. All 40 studies met the criteria for Q4-Q8, including adequate descriptions of subjects and settings (Q4), sufficient sample coverage (Q5), valid and reliable measurements (Q6, Q7), and appropriate statistical analysis (Q8). However, only 5 studies (12.5%) had an appropriate sampling frame (Q1), 2 studies (5.0%) used appropriate sampling methods (Q2), and 8 studies (20.0%) had adequate sample sizes (Q3). For Q9 (response rate), 22 studies (55.0%) met the criterion.

**Table 2:** Summary of methodological quality across included studies.

Item	N (%)
Appropriate sampling frame (Q1)	5 (12.5%)
Adequate sampling method (Q2)	2 (5.0%)
Adequate sample size (Q3)	8 (20.0%)
Detailed description of subjects and setting (Q4)	40 (100%)
Data analysis covering identified sample (Q5)	40 (100%)
Validated assessment tool or diagnostic criteria (Q6)	40 (100%)
Standard and reliable measurement (Q7)	40 (100%)
Appropriate statistical analysis (Q8)	40 (100%)
Adequate response rate or appropriately managed (Q9)	22 (55.0%)
Low risk of bias (≥ 70% “Yes”)	11 (27.5%)
Moderate risk of bias (50%–69% “Yes”)	26 (65.0%)
High risk of bias (≤ 49% “Yes”)	3 (7.5%)

### 3.3 Pooled prevalence of PD among critically ill children

**Figure 2:**
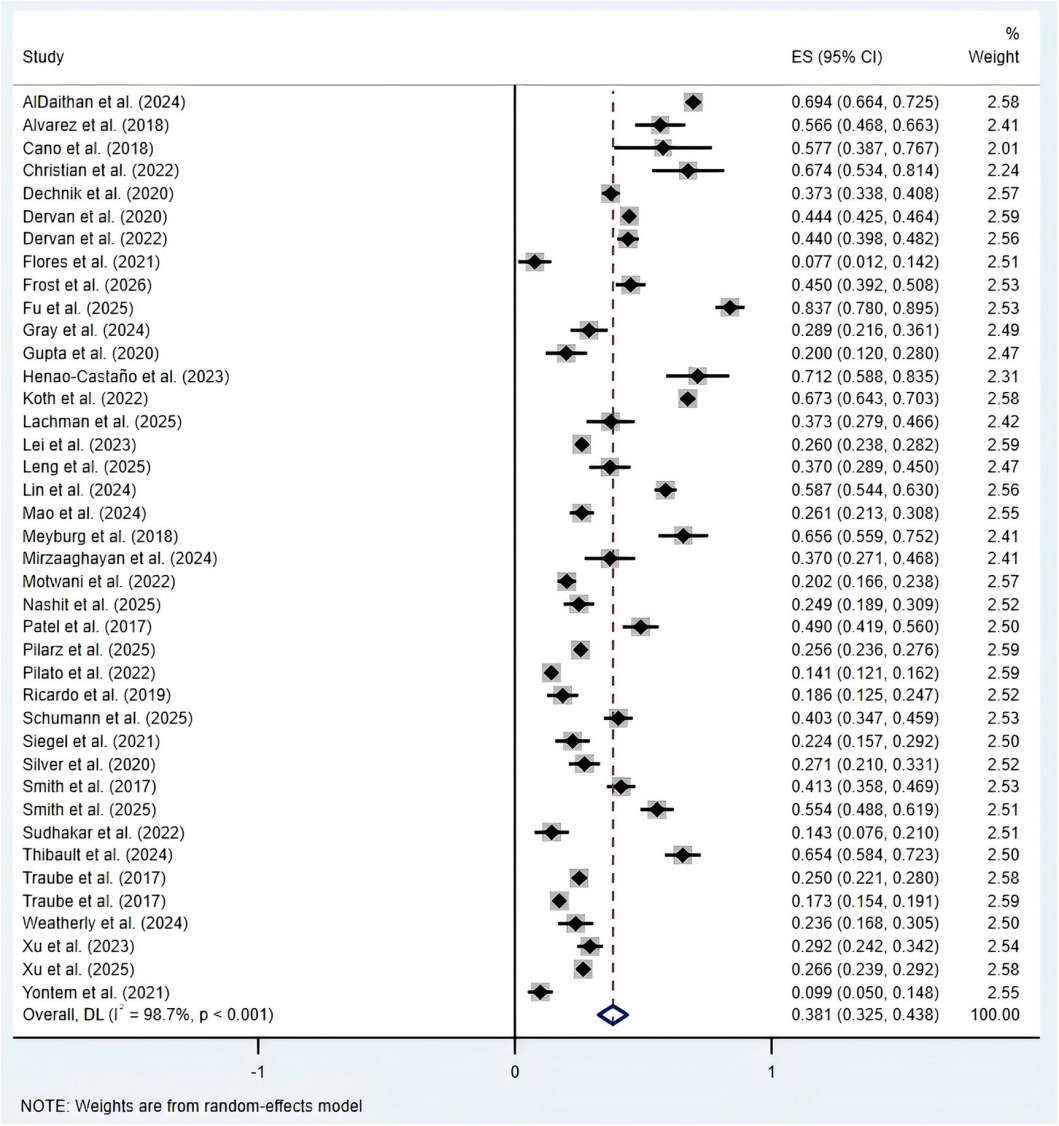
Forest plot of pooled prevalence of PD among critically ill children.

Given the substantial heterogeneity observed across studies, a random-effects model was applied to estimate the pooled prevalence (Figure 2). The meta-analysis revealed that the overall prevalence of PD in critically ill children was 38.1% (95% CI: 32.5–43.8%, I^2^ = 98.7%, 95% PI: 3.6–72.4%).

### 3.4 Subgroup analysis and meta-regression analysis

To investigate the sources of heterogeneity, we performed subgroup analyses based on income status, study design, and assessment tool (Table 3). As illustrated in Supplementary Figure S1, the pooled prevalence of PD was similar between high-income (40.9%, 95% CI: 33.5–48.3%) and middle-income countries (33.5%, 95% CI: 24.6–42.4%). In addition, stratification by study design (Supplementary Figure S2) showed comparable prevalence estimates for cohort studies (38.8%, 95% CI: 25.5–45.0%) and cross-sectional (35.7%, 95% CI: 21.0–50.5%). Subgroup analysis based on the assessment tool revealed a statistically significant difference in the pooled prevalence of PD (*P* = 0.017). Specifically, the highest prevalence was observed in studies using psCAM-ICU (51.1%, 95% CI: 38.4–63.9%), followed by CAPD (38.2%, 95% CI: 31.9–44.6%) and pCAM-ICU (27.7%, 95% CI: 17.6–37.7%).

**Table 3:** Subgroup analyses of the prevalence of PD among critically ill children.

Subgroups	Number of studies	Prevalence (95% CI)	Heterogeneity		*P* values across subgroups
			I²	*P* values	
Income status					0.211
High income	25	40.9% (33.5–48.3%)	98.9%	*P* < 0.001	
Middle income	15	33.5% (24.6–42.4%)	98.2%	*P* < 0.001	
Study design					0.709
Cohort	32	38.8% (25.5–45.0%)	98.8%	*P* < 0.001	
Cross-sectional	8	35.7% (21.0–50.5%)	98.3%	*P* < 0.001	
Assessment tool					0.017
CAPD	32	38.2% (31.9–44.6%)	98.9%	*P* < 0.001	
pCAM-ICU	5	27.7% (17.6–37.7%)	86.7%	*P* < 0.001	
psCAM-ICU	3	51.1% (38.4–63.9%)	89.4%	*P* < 0.001	

Univariate meta-regression analysis (Table 4) revealed no significant associations between the pooled prevalence of PD and any moderators, including sample size, income status, study design, and assessment tool (all *P* > 0.05).

**Table 4:** Meta-regression results.

Variables	Number of studies	Coefficient	Standard Error	95%CI	t values	*P* values
Sample size	40	−4.48 × 10-⁵	5.65 × 10-⁵	−0.0002–0.0001	−0.79	0.434
Income status (ref: High income)						
Middle income	26	−0.0752	0.0661	−0.2091–0.0587	−1.14	0.262
Study design (ref: Cohort)						
Cross-sectional	8	−0.0332	0.0842	−0.2039–0.1375	−0.39	0.696
Assessment tool (ref: CAPD)						
pCAM-ICU	5	−0.0905	0.0967	−0.2866–0.1056	−0.94	0.356
psCAM-ICU	3	0.1374	0.1201	−0.1062–0.3811	1.14	0.260

### 3.5 Publication bias and sensitivity analysis

**Figure 3:**
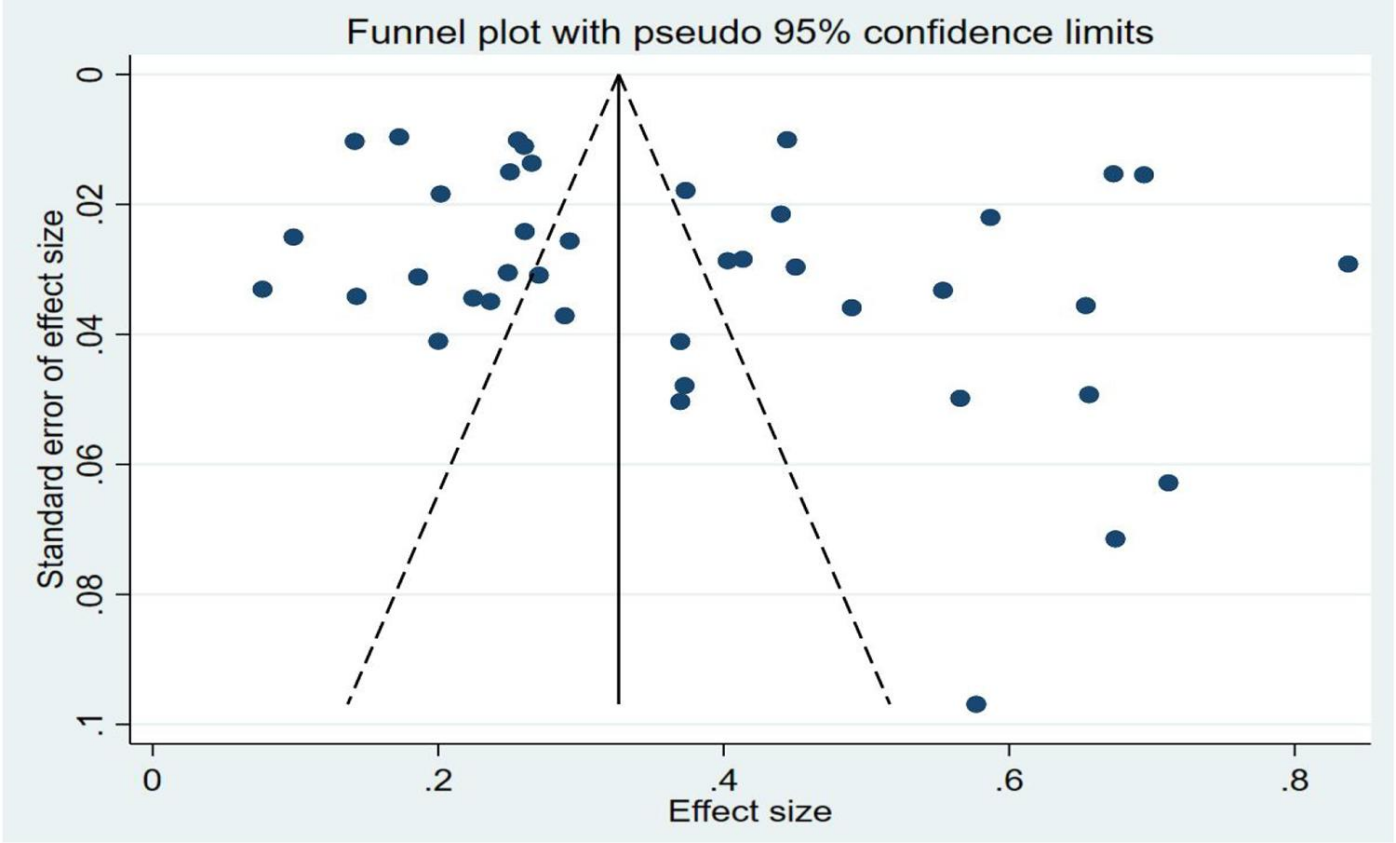
Funnel plot of prevalence of PD in critically ill children.

We assessed publication bias by visually examining the funnel plot (Figure 3) and performing Egger's test. The results showed no evidence of substantial publication bias (Egger's test: t = 1.67, *P* = 0.105; Supplementary Figure S4). To further evaluate the robustness of the pooled prevalence estimate, we performed several sensitivity analyses. First, a leave-one-out sensitivity analysis showed that no single study unduly influenced the overall estimate (Supplementary Table S4). The pooled prevalence after excluding each individual study ranged from 37.3% to 38.9%. Second, after excluding studies with a high risk of bias, the pooled prevalence was 37.4% (95% CI: 31.7–43.2%), similar to the main analysis (Supplementary Figure S5). Third, excluding very small studies did not materially change the estimate (36.1%, 95% CI:29.9–42.2%) (Supplementary Figure S6). Fourth, restricting the analysis to cohort studies only yielded a comparable prevalence of 38.8% (95% CI: 32.5–45.0%) (Supplementary Figure S7). Finally, when the analysis was limited to studies using the CAPD assessment tool, the pooled prevalence was 38.2% (95% CI: 31.9–44.6%) (Supplementary Figure S8). These sensitivity analyses confirmed the stability and robustness of our main findings.

## 4 Discussion

Given that PD is a debilitating and burdensome neurological complication in critically ill children, this meta-analysis was conducted to estimate its prevalence based on a pooled sample of 18,875 participants from 11 countries. With a pooled prevalence of 38.1%, this systematic review and meta-analysis demonstrated that PD affected a substantial proportion of critically ill children, a rate significantly exceeding that observed in pediatric general ward patients and critically ill adults (48, 49). However, the 95% PI for the true prevalence ranged from 3.6% to 72.4%, indicating that the prevalence of PD could vary substantially across different settings. This wide prediction interval, consistent with the high observed heterogeneity, suggests that while the average prevalence is approximately 38%, the true prevalence in a future study or a specific setting may fall anywhere within this broad range, depending on local clinical practices, patient case mix, sedation protocols, and delirium screening methods. Meanwhile, these findings may indicate that the pooled prevalence of 38.1% should not be interpreted as a stable universal estimate but rather as a summary of highly heterogeneous studies. Furthermore, the sources of heterogeneity remain insufficiently explained. Subgroup analysis suggested significant differences according to the assessment tool, with higher prevalence in studies using psCAM-ICU, followed by CAPD and pCAM-ICU. However, meta-regression did not identify significant associations with sample size, income status, study design, or assessment tool. This discrepancy indicates that the observed heterogeneity is not fully accounted for by the examined factors. In addition, the methodological quality of the included studies raises substantial concerns. Specifically, only 11 studies were classified as having a low risk of bias, whereas 26 were rated as moderate risk and 3 as high risk. Of greater concern, merely five studies employed an appropriate sampling frame, only two utilized suitable sampling methods, and only eight had an adequate sample size. These limitations warrant prominent consideration when interpreting the present findings. Therefore, although PD is undeniably a common complication in critically ill children, clinicians should be cautious when applying this pooled estimate to their context, and future research should focus on standardizing assessment tools and reporting key covariates to better understand the sources of this substantial variation. Understanding the underlying mechanisms of PD is crucial for developing targeted prevention and management strategies. The high prevalence of PD in critically ill children may be attributed to the vulnerability of the developing brain to physiological and iatrogenic disruption (50, 51), the frequent use of sedatives and analgesics that interfere with neurotransmitter balance (52, 53), and the psychological distress caused by separation from caregivers in an unfamiliar ICU environment (54, 55).

A critical finding of this meta-analysis is that the delirium assessment tool was the only subgroup variable that significantly correlated with the pooled prevalence estimates, highlighting the critical importance of understanding differences among these tools. The overall prevalence of PD varied substantially by assessment tool: highest with psCAM-ICU (51.1%), followed by CAPD (38.2%), and lowest with pCAM-ICU (27.7%). These tools differ not only in diagnostic thresholds and sensitivity but also in applicable age ranges, screening procedures, and clinical interpretation. Specifically, the pCAM-ICU, adapted from the CAM-ICU for pediatric use, focuses on acute changes in consciousness and attentional deficits (56). Although not specifically designed for this purpose, the pCAM-ICU has been shown in clinical studies to identify hypoactive delirium (34). However, its age applicability and screening algorithm may miss subtle presentations. In contrast, the CAPD employs an observational and interactive approach that captures a broader spectrum of delirium features including hypoactive subtypes, and it may be suitable across a wider age range, potentially explaining its higher detection rate (57). The psCAM-ICU, despite yielding the highest pooled prevalence, was examined in only three studies. Therefore, this estimate should be interpreted with caution, and its performance across different age groups and clinical settings remains to be validated. Taken together, these results underscore the absence of a standardized gold standard for pediatric delirium assessment and demonstrate that the heterogeneity in prevalence estimates is substantially driven by the choice of assessment tool. Future research should prioritize head-to-head comparisons of these tools across the same populations and standardize reporting to enable meaningful comparisons (58).

Stratified analysis by income status indicated that the pooled prevalence of PD in high-income countries appeared to be higher than in middle-income countries, although this finding was not statistically significant. The higher prevalence of PD in high-income countries may be due to more rigorous monitoring procedures, including routine delirium screening and standardized assessment practices, which facilitate the identification of positive cases. In addition, the greater availability and use of sedatives and analgesics in intensive care units in high-income countries may considerably increase the risk of delirium (59). Moreover, the lower prevalence observed in middle-income countries may reflect underdiagnosis rather than a lower actual incidence, particularly in the case of mild delirium, which tends to be more subtle and easily overlooked without systematic screening (60). It is also crucial to acknowledge the limited number of studies from middle-income countries, which might compromise the accuracy of prevalence estimates. These findings suggest that income-related disparities in the prevalence of PD may be influenced by the interplay of complex factors, including monitoring practices, treatment intensity, and reporting patterns, highlighting the need for implementing standardized assessment protocols across different healthcare settings. Moreover, comparable prevalence estimates were found in subgroup analyses of cohort studies and cross-sectional studies, with no significant difference between the two groups. This consistency implies that the findings of this meta-analysis are robust and not significantly affected by variations in study design.

## 5 Strengths and limitations

Notably, our study has several notable strengths. First, we explicitly restricted inclusion to studies with prevalence as a primary or clearly reported outcome. This approach reduces potential bias from study designs not originally intended to estimate prevalence. Second, the previous review did not conduct sensitivity analyses or publication bias assessments, which may compromise the reliability of its pooled estimates. In contrast, we performed leave-one-out analysis, multiple sensitivity analyses (excluding high-risk-of-bias studies, very small studies, and cross-sectional studies), and funnel plot and Egger's test. These analyses confirmed the robustness of our findings and showed no substantial publication bias. Third, this meta-analysis incorporates newly published prospective studies and includes data from 11 countries across different income levels, providing a more comprehensive synthesis of PD prevalence in critically ill children across geographic regions and healthcare resource settings. Fourth, we calculated the 95% prediction interval to illustrate the potential range of true prevalence in future settings and reinforces caution against overgeneralizing the point estimate.

Several limitations warrant consideration. First, in line with findings from other meta-analyses of prevalence studies, the included studies demonstrated considerable heterogeneity and a wide prediction interval. However, such heterogeneity was not fully explained in either subgroup or meta-regression analyses. In particular, the subgroup and meta-regression analyses involving the psCAM-ICU and pCAM-ICU were based on only a small number of studies, which may have limited the statistical power to detect significant associations. Therefore, the absence of statistically significant findings should not be interpreted as evidence that these factors do not contribute to heterogeneity. Therefore, the pooled prevalence of PD should not be interpreted as a stable universal estimate but rather as a summary of highly heterogeneous studies. Second, since the included studies primarily came from higher and middle-income countries, these findings may not be reliable for low-income countries. Third, studies published in languages other than English were excluded, which may introduce potential selection bias. This limitation is particularly important because our meta-analysis aims to synthesize global prevalence data and includes subgroup analyses by country income status. The exclusion of non-English studies may have skewed the pooled estimates and subgroup comparisons, potentially underrepresenting findings from low or middle income countries where non-English publications are more common. Although no significant publication bias was detected via funnel plots or the Egger test, its impact could not be fully ruled out. Fourth, several important clinical moderators (screening frequency, age, mechanical ventilation, sedative exposure, illness severity, developmental delay, ICU type, post-operative status, and delirium screening frequency) could not be explored due to insufficient reporting across studies. This major limitation prevents a nuanced understanding of heterogeneity and reduces clinical applicability. Future studies should systematically report these characteristics.

## 6 Conclusion

In conclusion, this meta-analysis indicates that PD is a common neurological complication in critically ill children. However, the extremely high heterogeneity and wide 95% PI substantially limit the interpretability of this estimate. Moreover, the methodological quality of the included studies raises substantial concerns, as only a minority were rated as low risk of bias, and few had appropriate sampling frames or adequate sample sizes. Therefore, the pooled prevalence should not be interpreted as a stable universal estimate but rather as a summary of highly heterogeneous studies. Future research should prioritize high-quality study designs, standardized assessment tools, and systematic reporting of key covariates to better understand the sources of heterogeneity. These results both underscore the importance of standardized PD assessments in critically ill children and highlight the need for high-quality interventional studies.

## Data Availability

The original contributions presented in the study are included in the article/Supplementary Material, further inquiries can be directed to the corresponding author.
