# Supplementary material for: Prevalence of pediatric delirium among critically ill children: a meta-analysis of observational studies
Source: Front Pediatr. 2026 Jul 22;14:1888789. doi: 10.3389/fped.2026.1888789 (PMC13437776; doi:10.3389/fped.2026.1888789)
Supplement: Supplementary file 1 [file Table1.docx]

**Supplemental Materials**

Table S1. The search strategies.

| Databases | Step | Search Strategies |
| --- | --- | --- |
| PubMed | #1 | "intensive care units, pediatric"[Mesh Terms] Sort by: Best match |
|  | #2 | "critical illness"[Mesh Terms] Sort by: Best match |
|  | #3 | "critically ill child"[Title/Abstract] OR "child"[Title/Abstract] OR "infant"[Title/Abstract] OR "adolescent"[Title/Abstract] OR "critically ill children"[Title/Abstract] OR "critically ill pediatric patients"[Title/Abstract] OR "pediatric intensive care units"[Title/Abstract] OR "PICU"[Title/Abstract] Sort by: Best match |
|  | #4 | "delirium"[Mesh Terms] Sort by: Best match |
|  | #5 | "confusion"[Mesh Terms] Sort by: Best match |
|  | #6 | "delirium"[Title/Abstract] OR "pediatric delirium"[Title/Abstract] OR "confusion"[Title/Abstract] OR "encephalopathy"[Title/Abstract] OR "acute confusion"[Title/Abstract] OR "acute brain dysfunction"[Title/Abstract] OR "ICU delirium"[Title/Abstract] Sort by: Best match |
|  | #7 | "prevalence"[Mesh Terms] Sort by: Best match |
|  | #8 | "incidence"[Mesh Terms] Sort by: Best match |
|  | #9 | "e[pidemiology](https://www.ncbi.nlm.nih.gov/mesh/68004813)"[Mesh Terms] Sort by: Best match |
|  | #10 | "prevalence"[Title/Abstract] OR "incidence"[Title/Abstract] OR "e[pidemiology](https://www.ncbi.nlm.nih.gov/mesh/68004813)"[Title/Abstract] Sort by: Best match |
|  | #11 | #1 OR #2 OR #3 Sort by: Best match |
|  | #12 | #4 OR #5 OR #6 Sort by: Best match |
|  | #13 | #7 OR #8 OR #9 OR #10 Sort by: Best match |
|  | #14 | #11 AND #12 AND #13 Sort by: Best match |
| Web of Science | #1 | **Ts=(**critically ill child**) OR TS=(**child) **OR TS=(**infant) **OR TS=(**adolescent) **OR TS=(**critically ill children) OR TS=(critically ill pediatric patients) OR Ts=(pediatric intensive care units) **OR TS=(PICU**) |
|  | #2 | **Ts=(delirium) OR Ts=(pediatric delirium) OR Ts=(confusion) OR** TS=(encephalopathy) OR **TS=(**acute confusion) OR **TS=(**acute brain dysfunction) OR **TS=(ICU delirium**) |
|  | #3 | TS=(prevalence) OR **TS=(incidence) OR TS=(**e[pidemiology](https://www.ncbi.nlm.nih.gov/mesh/68004813)) |
|  | #4 | #1 AND #2 AND #3 |
| Scopus | #1 | TITLE-ABS-KEY ("critically ill child" OR "child" OR "infant" OR "adolescent" OR "critically ill children" OR "critically ill pediatric patients" OR "pediatric intensive care units" OR "PICU") |
|  | #2 | TITLE-ABS-KEY ("delirium" OR "**pediatric delirium**" OR "confusion" OR "encephalopathy" OR "acute confusion" OR "acute brain dysfunction" OR "ICU delirium") |
|  | #3 | TITLE-ABS-KEY ("prevalence" OR "incidence" OR "e[pidemiology](https://www.ncbi.nlm.nih.gov/mesh/68004813)") |
|  | #4 | #1 AND #2 AND #3 |
| Embase | **#1** | 'critically ill child**':ti,ab,kw OR '**child**':ti,ab,kw OR '**infant**':ti,ab,kw OR '**adolescent**':ti,ab,kw OR '**critically ill children**':ti,ab,kw OR '**critically ill children**':ti,ab,kw OR '**critically ill pediatric patients**':ti,ab,kw OR '**pediatric intensive care units**':ti,ab,kw OR 'PICU':ti,ab,kw** |
|  | **#2** | 'delirium**':ti,ab,kw OR 'pediatric delirium':ti,ab,kw OR 'confusion':ti,ab,kw OR '**encephalopathy**':ti,ab,kw OR '**acute confusion**':ti,ab,kw OR '**acute brain dysfunction**':ti,ab,kw OR 'ICU delirium':ti,ab,kw** |
|  | **#3** | 'prevalence**':ti,ab,kw OR 'incidence':ti,ab,kw OR '**e[pidemiology](https://www.ncbi.nlm.nih.gov/mesh/68004813)**':ti,ab,kw** |
|  | **#4** | #1 AND #2 AND #3 |
| Cochrane Library | #1 | (critically ill child)**:ti,ab,kw OR (**child)**:ti,ab,kw OR (**infant)**:ti,ab,kw OR (**adolescent)**:ti,ab,kw OR (**critically ill children)**:ti,ab,kw OR (**critically ill pediatric patients)**:ti,ab,kw OR (**pediatric intensive care units)**:ti,ab,kw OR (PICU):ti,ab,kw** |
|  | #2 | Mesh descriptor: [Delirium] explode all trees |
|  | #3 | (delirium)**:ti,ab,kw OR (pediatric delirium**)**:ti,ab,kw OR (confusion**)**:ti,ab,kw OR (**encephalopathy)**:ti,ab,kw OR (**acute confusion)**:ti,ab,kw OR (**acute brain dysfunction)**:ti,ab,kw OR (ICU delirium**)**:ti,ab,kw** |
|  | #4 | (prevalence)**:ti,ab,kw OR (incidence**)**:ti,ab,kw OR (**e[pidemiology](https://www.ncbi.nlm.nih.gov/mesh/68004813))**:ti,ab,kw** |
|  | #5 | #2 OR #3 |
|  | #6 | #1 AND #4 AND #5 |
| CINAHL | S1 | TI critically ill child **OR TI** child **OR TI** infant **OR TI** adolescent **OR TI** critically ill children OR TI critically ill pediatric patients OR TI pediatric intensive care units **OR TI PICU** |
|  | S2 | **TI delirium OR TI pediatric delirium OR TI confusion OR** TI encephalopathy OR **TI** acute confusion |
|  | S3 | TI prevalence OR **TI incidence OR TI** e[pidemiology](https://www.ncbi.nlm.nih.gov/mesh/68004813) |
|  | S4 | S1 AND S2 AND S3 |

Table S2. Excluded full‑text studies and specific reasons for exclusion.

| **Citation** | **Reason for exclusion** |
| --- | --- |
| Ge, X. H., Wei, W. R., Feng, T. N., Xu, L. L., Hu, Y. Q., & Yuan, C. R. (2021). Analysis of risk factor for pediatric intensive care unit delirium in children: a case-control study. American journal of translational research, 13(8), 9143–9151. | Study design is inappropriate (Case-control study) |
| Xu, T. T., Zhang, Y. C., Ye, X. F., Fu, C. H., Li, Y., Ju, M. J., Liu, J., Yang, X. Y., & Zhang, W. Y. (2023). Risk factors of delirium in a paediatric intensive care unit: A prospective case series study. Nursing in critical care, 28(5), 645–652. <https://doi.org/10.1111/nicc.12920> | Study design is inappropriate (Case series study) |
| Kolmar, A. R., Paton, A. M., Kramer, M. A., & Guilliams, K. P. (2024). Differences in Delirium Evaluation and Pharmacologic Management in Children With Developmental Delay: A Retrospective Case-Control Study. Journal of intensive care medicine, 39(2), 170–175. <https://doi.org/10.1177/08850666231194534> | Study design is inappropriate (Case-control study) |
| Di Nardo, M., Boldrini, F., Broccati, F., Cancani, F., Satta, T., Stoppa, F., Genuini, L., Zampini, G., Perdichizzi, S., Bottari, G., Fischer, M., Gawronski, O., Bonetti, A., Piermarini, I., Recchiuti, V., Leone, P., Rossi, A., Tabarini, P., Biasucci, D., Villani, A., … Choong, K. (2021). The LiberAction Project: Implementation of a Pediatric Liberation Bundle to Screen Delirium, Reduce Benzodiazepine Sedation, and Provide Early Mobilization in a Human Resource-Limited Pediatric Intensive Care Unit. Frontiers in pediatrics, 9, 788997. https://doi.org/10.3389/fped.2021.788997 | Study design is inappropriate (Interventional study) |
| Schieveld, J. N., Leroy, P. L., van Os, J., Nicolai, J., Vos, G. D., & Leentjens, A. F. (2007). Pediatric delirium in critical illness: phenomenology, clinical correlates and treatment response in 40 cases in the pediatric intensive care unit. Intensive care medicine, 33(6), 1033–1040. <https://doi.org/10.1007/s00134-007-0637-8> | Study design is inappropriate (Case series study) |
| Dornette, S., Deptola, S., Hemmann, B., Venkatesan, C., & Cortezzo, D. E. (2025). Recognition and Management of Delirium in the Neonatal Intensive Care Unit: Case Series From a Single-Center Level 4 Intensive Care Unit. Journal of child neurology, 40(1), 67–70. https://doi.org/10.1177/08830738241282099 | Study design is inappropriate (Case series study) |
| Jesus, A. O., Jones, L., Linares, R., Buck, M. L., & Frank, D. U. (2020). Management of Hyperactive Delirium in the Pediatric Intensive Care Unit: Case Series of Three Young Children. Journal of pediatric intensive care, 9(2), 119–123. https://doi.org/10.1055/s-0039-3400458 | Study design is inappropriate (Case series study) |
| Patel, A. K., Biagas, K. V., Clark, E. C., & Traube, C. (2017). Delirium in the Pediatric Cardiac Extracorporeal Membrane Oxygenation Patient Population: A Case Series. Pediatric critical care medicine : a journal of the Society of Critical Care Medicine and the World Federation of Pediatric Intensive and Critical Care Societies, 18(12), e621–e624.https://doi.org/10.1097/PCC.0000000000001364 | Study design is inappropriate (Case series study) |
| Azamfirei, R., Behrens, D., Padilla, S., Madden, K., Goldberg, S., Geno, M., Manning, M. J., Piole, M., Madsen, E., Maue, D., Abu-Sultaneh, S., Awojoodu, R., Wang, N. Y., Needham, D. M., Neufeld, K., & Kudchadkar, S. R. (2024). Delirium Screening in Critically Ill Children: Secondary Analysis of the Multicenter PICU Up! Pilot Trial Dataset, 2019-2020. Pediatric critical care medicine : a journal of the Society of Critical Care Medicine and the World Federation of Pediatric Intensive and Critical Care Societies, 25(10), 880–888. https://doi.org/10.1097/PCC.0000000000003555 | Lack of sufficient data |
| Nelligan, M., Nellis, M. E., Mauer, E. A., Gerber, L. M., & Traube, C. (2023). Association between Platelet Transfusion and Delirium in Critically Ill Children. Children (Basel, Switzerland), 10(5), 825. https://doi.org/10.3390/children10050825 | Lack of sufficient data |
| Silver, G., Traube, C., Gerber, L. M., Sun, X., Kearney, J., Patel, A., & Greenwald, B. (2015). Pediatric delirium and associated risk factors: a single-center prospective observational study. Pediatric critical care medicine : a journal of the Society of Critical Care Medicine and the World Federation of Pediatric Intensive and Critical Care Societies, 16(4), 303–309. https://doi.org/10.1097/PCC.0000000000000356 | Lack of sufficient data |
| Traube, C., Mauer, E. A., Gerber, L. M., Kaur, S., Joyce, C., Kerson, A., Carlo, C., Notterman, D., Worgall, S., Silver, G., & Greenwald, B. M. (2016). Cost Associated With Pediatric Delirium in the ICU. Critical care medicine, 44(12), e1175–e1179. https://doi.org/10.1097/CCM.0000000000002004 | Lack of sufficient data |
| Siegel, E. J., & Traube, C. (2020). Pediatric delirium: epidemiology and outcomes. Current opinion in pediatrics, 32(6), 743–749. https://doi.org/10.1097/MOP.0000000000000960 | Lack of sufficient data |
| Lyu J, Jia Y, Yan M, et al. Risk factors for postoperative delirium in children with congenital heart disease: a prospective nested case-control study. 先天性心脏病患儿术后谵妄危险因素的前瞻性巢式病例对照研究. Zhongguo Dang Dai Er Ke Za Zhi. 2022;24(3):232-239. doi:10.7499/j.issn.1008-8830.2110026 | Non-English |
| Yu, X., Wang, L., Gao, Y., Xie, Z. X., & Li, G. (2022). 惊厥患儿镇静后谵妄发生的危险因素及风险列线图预测模型的建立 [Risk factors for delirium after sedation in children with convulsion and establishment of a nomogram model for predicting the risk of delirium]. Zhongguo dang dai er ke za zhi = Chinese journal of contemporary pediatrics, 24(11), 1238–1245. https://doi.org/10.7499/j.issn.1008-8830.2205076 | Non-English |

Table S3. Quality assessment results of included studies.

| Study | Q1 | Q2 | Q3 | Q4 | Q5 | Q6 | Q7 | Q8 | Q9 | Overall |
| --- | --- | --- | --- | --- | --- | --- | --- | --- | --- | --- |
| AlDaithan et al. (2024) | N | N | U | Y | Y | Y | Y | Y | Y | M |
| Alvarez et al. (2018) | N | N | U | Y | Y | Y | Y | Y | Y | M |
| Cano et al. (2018) | N | N | N | Y | N | Y | Y | Y | N | H |
| Christian et al. (2022) | N | N | U | Y | Y | Y | Y | Y | U | M |
| Dechnik et al. (2020) | N | N | U | Y | Y | Y | Y | Y | N | M |
| Dervan et al. (2020) | Y | U | Y | Y | Y | Y | Y | Y | Y | L |
| Dervan et al. (2022) | N | N | U | Y | Y | Y | Y | Y | Y | M |
| Flores et al. (2021) | N | N | Y | Y | Y | Y | Y | Y | Y | L |
| Frost et al. (2026) | N | N | U | Y | Y | Y | Y | Y | Y | M |
| Fu et al. (2025) | N | N | Y | Y | Y | Y | Y | Y | Y | L |
| Gray et al. (2024) | N | N | U | Y | Y | Y | Y | Y | U | M |
| Gupta et al. (2020) | N | N | U | Y | Y | Y | Y | Y | U | M |
| Henao-Castaño et al. (2023) | N | N | Y | Y | Y | Y | Y | Y | U | M |
| Koth et al. (2022) | N | N | U | Y | Y | Y | Y | Y | U | M |
| Lachman et al. (2025) | N | N | U | Y | Y | Y | Y | Y | N | M |
| Lei et al. (2023) | N | N | Y | Y | Y | Y | Y | Y | Y | L |
| Leng et al. (2025) | N | N | U | Y | Y | Y | Y | Y | Y | M |
| Lin et al. (2024) | N | N | Y | Y | Y | Y | Y | Y | Y | L |
| Mao et al. (2024) | N | N | U | Y | Y | Y | Y | Y | Y | M |
| Meyburg et al. (2018) | N | N | U | Y | Y | Y | Y | Y | Y | M |
| Mirzaaghayan et al. (2024) | N | N | Y | Y | Y | Y | Y | Y | U | M |
| Motwani et al. (2022) | N | N | U | Y | Y | Y | Y | Y | U | M |
| Nashit et al. (2025) | N | N | U | Y | Y | Y | Y | Y | Y | M |
| Patel et al. (2017) | N | N | U | Y | U | Y | Y | Y | U | H |
| Pilarz et al. (2025) | Y | N | Y | Y | Y | Y | Y | Y | Y | L |
| Pilato et al. (2022) | N | N | U | Y | Y | Y | Y | Y | U | M |
| Ricardo et al. (2019) | N | N | Y | Y | Y | Y | Y | Y | Y | M |
| Schumann et al. (2025) | N | N | Y | Y | Y | Y | Y | Y | Y | M |
| Siegel et al. (2021) | N | N | U | Y | Y | Y | Y | Y | Y | M |
| Silver et al. (2020) | N | N | U | Y | Y | Y | Y | Y | N | M |
| Smith et al. (2017) | N | N | U | Y | Y | Y | Y | Y | U | M |
| Smith et al. (2025) | Y | Y | Y | Y | Y | Y | Y | Y | U | L |
| Sudhakar et al. (2022) | N | N | U | Y | Y | Y | Y | Y | Y | M |
| Thibault et al. (2024) | N | N | U | Y | Y | Y | Y | Y | Y | M |
| Traube et al. (2017) | Y | N | Y | Y | Y | Y | Y | Y | Y | L |
| Traube et al. (2017) | Y | N | Y | Y | Y | Y | Y | Y | Y | L |
| Weatherly et al. (2024) | N | N | U | Y | Y | Y | Y | Y | Y | M |
| Xu et al. (2023) | N | N | U | Y | Y | Y | Y | Y | Y | M |
| Xu et al. (2025) | Y | N | Y | Y | Y | Y | Y | Y | Y | L |
| Yontem et al. (2021) | N | N | U | Y | Y | Y | Y | Y | Y | M |

Notes: Y: Yes, N: No, U: Unclear. L: Low risk, M: Moderate risk, H: High risk.

Q1: Was the sample frame appropriate to address the target population?

Q2: Were study participants sampled in an appropriate way?

Q3: Was the sample size adequate?

Q4: Were the study subjects and the setting described in detail?

Q5: Was the data analysis conducted with sufficient coverage of the identified sample?

Q6: Were valid methods used for the identification of the condition?

Q7: Was the condition measured in a standard, reliable way for all participants?

Q8: Was there appropriate statistical analysis?

Q9: Was the response rate adequate, and if not, was the low response rate managed appropriately?

Each study was categorized by risk of bias as follows: high risk if ≤ 49% of items are responded to with "yes;" moderate risk if 50%-69% of items are responded to with "yes; "and low risk if ≥ 70% of items are responded to with "yes."

Figure S1. The pooled prevalence of delirium in critically ill children based on income status.

Figure S2. The pooled prevalence of pediatric delirium in critically ill children based on study design.

Figure S3. The pooled prevalence of delirium in critically ill children based on assessment tool.

Figure S4. Egger's test results.

Table S4. The results of the sensitivity analysis.

| **Omitted Study** | **Prevalence** | **95% CI** |
| --- | --- | --- |
| AlDaithan et al. (2024) | 0.373 | 0.320-0.425 |
| Alvarez et al. (2018) | 0.377 | 0.320-0.434 |
| Cano et al. (2018) | 0.377 | 0.320-0.434 |
| Christian et al. (2022) | 0.375 | 0.318-0.432 |
| Dechnik et al. (2020) | 0.382 | 0.324-0.440 |
| Dervan et al. (2020) | 0.380 | 0.321-0.439 |
| Dervan et al. (2022) | 0.380 | 0.322-0.437 |
| Flores et al. (2021) | 0.389 | 0.332-0.446 |
| Frost et al. (2026) | 0.380 | 0.322-0.437 |
| Fu et al. (2025) | 0.369 | 0.315-0.424 |
| Gray et al. (2024) | 0.384 | 0.327-0.441 |
| Gupta et al. (2020) | 0.386 | 0.329-0.443 |
| Henao-Castaño et al. (2023) | 0.374 | 0.317-0.430 |
| Koth et al. (2022) | 0.373 | 0.320-0.426 |
| Lachman et al. (2025) | 0.382 | 0.324-0.439 |
| Lei et al. (2023) | 0.385 | 0.326-0.444 |
| Leng et al. (2025) | 0.382 | 0.324-0.439 |
| Lin et al. (2024) | 0.376 | 0.320-0.432 |
| Mao et al. (2024) | 0.385 | 0.327-0.442 |
| Meyburg et al. (2018) | 0.375 | 0.318-0.431 |
| Mirzaaghayan et al. (2024) | 0.382 | 0.325-0.439 |
| Motwani et al. (2022) | 0.386 | 0.329-0.444 |
| Nashit et al. (2025) | 0.385 | 0.328-0.442 |
| Patel et al. (2017) | 0.379 | 0.322-0.436 |
| Pilarz et al. (2025) | 0.385 | 0.325-0.445 |
| Pilato et al. (2022) | 0.388 | 0.331-0.444 |
| Ricardo et al. (2019) | 0.386 | 0.329-0.444 |
| Schumann et al. (2025) | 0.381 | 0.323-0.438 |
| Siegel et al. (2021) | 0.385 | 0.328-0.443 |
| Silver et al. (2020) | 0.384 | 0.327-0.442 |
| Smith et al. (2017) | 0.381 | 0.323-0.438 |
| Smith et al. (2025) | 0.377 | 0.320-0.434 |
| Sudhakar et al. (2022) | 0.388 | 0.330-0.445 |
| Thibault et al. (2024) | 0.374 | 0.318-0.431 |
| Traube et al. (2017) | 0.385 | 0.327-0.443 |
| Traube et al. (2017) | 0.387 | 0.329-0.444 |
| Weatherly et al. (2024) | 0.385 | 0.328-0.442 |
| Xu et al. (2023) | 0.384 | 0.326-0.441 |
| Xu et al. (2025) | 0.385 | 0.326-0.443 |
| Yontem et al. (2021) | 0.389 | 0.332-0.446 |

Figure S5. Sensitivity analysis excluding high risk of bias studies.

Figure S6. Sensitivity analysis excluding very small studies.

Figure S7. Sensitivity analysis excluding cross‑sectional studies.

Figure S8. Sensitivity analysis restricted to CAPD‑based studies.
